# A new paradigm for teaching behavior change: Implications for residency training in family medicine and psychiatry

**DOI:** 10.1186/1472-6920-12-64

**Published:** 2012-08-03

**Authors:** A Catalina Triana, Michael M Olson, Dorothy B Trevino

**Affiliations:** 1 Family Medicine Department, University of Texas Medical Branch, 301 University Boulevard, Galveston, TX 77555-1123, USA

**Keywords:** Primary care training, Counseling skills, Psychiatry training, Motivational interviewing, Curriculum, Teaching method

## Abstract

**Background:**

Primary care physicians (PCPs) provide ~50 % of all mental health services in the U.S. Given the widening gap between patient mental health needs and resources available to meet those needs, there is an increasing demand for family medicine and psychiatry trainees to master competencies in both behavioral medicine and primary care counseling during residency-if for no other reason than to accommodate the realities of medical practice given the oft present gap between the need for psychiatric services and the availability, quality, and/or affordability of specialized psychiatric care. To begin to address this gap, a skills-based, interactive curriculum based on motivational interviewing (MI) as a teaching method is presented.

**Methods:**

The curriculum described in this paper is a four-week block rotation taught in the second year of residency. Motivational interviewing (MI) is used as a teaching approach toward the goal of clinical behavior change. Residents’ strengths, personal choice and autonomy are emphasized. Each week of the rotation, there is a clinical topic and a set of specific skills for mastery. Residents are offered a “menu” of skills, role modeling, role/real play, practice with standardized patients (SP), and direct supervision in clinic.

**Results:**

Thirty-nine residents have completed the curriculum. Based on residents’ subjective reporting using pre-post scales (i.e., importance and confidence), all participants to date have reported substantial increases in confidence/self-efficacy using primary care counseling skills in their continuity clinic.

**Conclusions:**

This paper presents an innovative, empirically based model for teaching the essential skills necessary for physicians providing care for patients with mental/emotional health needs as well as health-behavior change concerns. Implications for training in the broader context, particularly as it relates to multi-disciplinary and collaborative models of teaching/training are discussed.

## Background

The global burden of untreated mental, neurological and substance abuse disorders is well documented in the literature as is the discussion of gaps in resources and treatment with neuropsychiatric disorders surpassing other disorders as the number one cause of disability [1-3]. The problem and scope of unmet mental health needs in the current health care delivery system is complex and multi-factorial and beyond the scope of this paper to detail in earnest. One key aspect however, as cited in a recent World Health Organization (WHO) publication, involves primary health care doctors not being properly equipped to manage mild and moderate mental disorders [2].

Family medicine and psychiatry residency programs (among others) collectively struggle to optimally train physicians to meet the burgeoning mental health need in this country. In psychiatry residency training, a few contemporary issues discussed in the literature include, 1) training adequate numbers of residents to meet the needs of the population [4], 2) an evolving dominance of psychobiologic over psychodynamic influences on education and practice [5-7], and 3) the need for institutional support for psychotherapy training, particularly in light of recent residency review committee (RRC) for Psychiatry requirements to demonstrate competency in psychotherapy [8].

Several studies have estimated that primary care physicians (PCP) provide almost half of all mental health services in the United States [9]. Patients with mental health problems are more likely to present with somatic complaints than with psychological/emotional symptoms to their PCP [1]. Counseling provided by family physicians has been shown to be both efficient and cost effective [9-11] and is the recommended starting point in a stepped-care approach [12]. In addition to providing help to patients with problems like depression and anxiety, family physicians routinely provide counseling to promote lifestyle changes such as smoking cessation, medication adherence, healthy diet and regular exercise [11,12]. The rates of chronic illness and disease related to health behaviors are on the rise among the US population, underscoring the need for effective counseling skills. This need is enhanced by recent trends in the field of Psychiatry with fewer medical students choosing psychiatry for residency, smaller numbers of practitioners, changes/shifts in state and federal equiparity to support provision of mental health/psychiatric services by psychiatrists, and perceived stigma by patients who are often more willing to visit with their PCP than a psychiatrist. If this trend continues, the burden on PCPs to be a point of access for patient for behavioral, mental, and emotional needs will only increase.

Despite the critical population need for primary mental health care, research shows that primary care residents frequently lack counseling skills and the confidence to apply them [13,14]. Time limitations and challenges arising from the health care delivery system are frequent barriers mentioned in the literature [14,15]. Resistance/ambivalence from learners to behavioral medicine skills and topics is also commonly encountered by teachers. This “resistance” may be partly influenced by the broader culture and philosophy of medicine that continues to be steeped in a fragmented or dualistic vs. a *cura personalis* or whole person approach that is non-Cartesian. Resistance may also be a function of low self-efficacy/confidence in applying the skills being taught or perceived relevance to practice.

Both the American Academy of Family Physicians (AAFP) and the World Organization of Family Doctors (WONCA) recognize mental health care as a core aspect of primary care training [1] and practice [16]. Despite this recognition, mental health and psychiatry training in primary care residency programs varies significantly in quantity and quality [17] with concern being raised that many current teaching methods in behavioral health are ultimately ineffective in changing actual clinical practice patterns [18]. There is a paucity of published curricula on mental health care for PCPs in the literature. The few published reports to date have utilized a wide variety of content, methodology, and evaluation measures. Taken together, existing findings are tentative at best. However, according to Hodges (2001), the variables that appear most critical are “duration of the intervention, the degree of active participation of the learners, and the degree of integration of new learning in the learners’ clinical context [19].” These authors further contend that existing evidence point to the need for ongoing, interactive and contextually relevant mental health training for PCPs. Despite the lack of empirical direction, residencies continue to struggle with the important issue of providing adequate training in mental health care/psychiatry in primary care. Findings from a more recent survey of program directors revealed that a vast majority desired more training in mental health care and psychiatry for primary care and recognized that PCP’s should be ready and willing to treat more psychiatric conditions [17].

A key finding from the research literature that has applicability to both family medicine and psychiatry residencies is that change in practice behavior(s) is largely determined by levels of confidence or self-efficacy [20-24]. In light of these data and concerns raised by national accrediting bodies, the need for innovative, empirically-driven, interactive, and contextually relevant mental health training for primary care physicians is apparent [19]. In particular, the training to meet primary care mental and behavioral health needs must be delivered and utilize a process that will increase the likelihood of integration of those skills into clinical practice [18], largely determined by residents’ self-efficacy or confidence.

## Methods

### Pilot curriculum

To address the need for innovative training that would increase the likelihood of integration of skills into clinical practice, our team developed a four-week behavioral medicine block rotation (BMR) for second year family medicine residents grounded in the latest developments/science in behavior change, using motivational interviewing (MI) as the basis for teaching. The key aim was to integrate the same MI processes into teaching that have been shown to help others change in clinical settings. This approach included content that was; 1) evidence-based, 2) contextually sensitive (applicable/relevant to actual clinical practice-authentic performance), and 3) engaging (learner centered). Motivational Interviewing has increasingly gained acceptance in primary care as an evidence-based, effective intervention for lifestyle changes, in particular as a brief intervention for substance abuse. A recent systematic review of MI training in primary care shows that the most frequent focus topic was diabetes, followed by counseling on alcohol and smoking. The studies reviewed showed heterogeneity in duration, focus and training methodology with overall favorable training outcomes [25]. Although the focus of this paper is on the block-rotation, this four-week experience is embedded in a three year longitudinal curriculum for family medicine residents and is beyond the scope of this paper to fully detail but is available upon request^a^.

Motivational Interviewing is an evidence-based, patient-centered counseling approach to elicit behavior change [26]. It emphasizes respect, the use of a non-judgmental attitude, and support of self-efficacy. We translated these central skills and tenants of (MI) into a learner- centered teaching strategy emphasizing residents’ strengths, personal choice and autonomy. The objective of using MI as a guiding approach to teaching was to increase residents’ confidence/self-efficacy and thereby increase the likelihood of clinical behavior change using these skills in practice.

Learner-centered teaching implies flexibility in the content being delivered, an understanding of the context wherein the skills are being applied, and sensitivity to the developmental level or “place” of the learner. For example, some of the particular needs of the residents being taught in our program included; ability to integrate skills into a time-limited visit (i.e. 15–20 minutes), practice management issues (e.g., billing, coding, documentation), and the need for direct observation, modeling, and feedback in practice.

### Process

On average, three to four residents participate in the BMR each time. The BMR is facilitated by a behavioralist and a family doctor. This multi-disciplinary team approach is intended to model collaboration and is a parallel process with residents collaborating with peers/colleagues and with patients as they provide clinical care.

Each week, a progressive set of skills with content determined to be of highest relevance to our learners (e.g, depression, anxiety, primary care counseling skills for behavior change) are covered. In week one, the focus is on general counseling skills and reflective listening; week two, health behavior change; week three, counseling for depression; and week four, counseling for anxiety. The objectives for each week of the rotation are listed in Table 1. The process of delivering the content throughout these four weeks is fully immersed in the core tenants and approach of motivational interviewing including; reflective listening, assessing importance and confidence using skills, rolling with/coming along side resistance, and emphasizing autonomy and personal-choice.

**Table 1:** Specific BMR Objectives by Week

	**Knowledge**	**Skills**	**Attitudes**
	**The resident will demonstrate understanding of:**	**The resident will integrate into a clinical interview:**	**The resident will display:**
Week 1	· General principles of effective communication	· Open ended questions	· Guiding vs. imparting communication style
MI I: General	· Stress and its influence in health	· Affirmations	
Counseling		· Reflective listening	
Skills		· Summaries	
		· Clinical feedback	
		· Offering Advice	
		· Assessment of stress	
Week 2	· Theoretical framework for health behavior change	· Assessment of importance	· Respect for patient’s autonomy
MI II: Health Behavior Change	· Evidence of usefulness of MI	· Assessment of confidence	
		· Scaling questions	
		· Explore costs and benefits of change	
		· Evocative questions	
Week 3	· DSM-IV criteria for MDD	· Screening for depression (PhQ9)	· Empathy
MI for Depression	· Differential diagnosis for depressive disorders	· Diagnosis of depression	
	· CBT theoretical background	· Psychoeducation on depression	
	Therapeutic guidelines	· Behavioral activation	
		· Pleasure list	
Week 4	· DSMIV criteria for GAD, Panic Disorder	· Screening for anxiety	· Awareness of mind-body connections
MI for anxiety	· Differential diagnosis for anxiety disorders	· Diagnosis of anxiety	
	· CBT theoretical background	· CBT techniques	
	Therapeutic guidelines	· Thought challenging	
		· Deep breathing	
		· Thought stopping	
		· Psychoeducation on anxiety	

To be clear, the significance of this approach is the use of MI as a teaching methodology throughout the four week rotation experience to facilitate residents’ learning and behavior change clinically. At the same time, residents/learners are learning to use these skills to interact with and counsel their patients utilizing the same evidence-based approach.

### Educational strategies

Educational strategies include: workshops, role/real-play exercises, standardized patient (SP) practice with feedback, and direct observation of residents by faculty in their continuity clinic, independent study, and a wellness project. For the wellness project, residents are offered a menu of ideas for health/well-being to which they add their own ideas. Each resident chooses a focus for themselves and time is allotted during the four weeks of the rotation for self-directed change. The wellness exercise/project models the process of health behavior change counseling but more importantly gets the learners closer to the lived experience of patients working toward a change goal.

Facilitated workshops start with a presentation of the list of skills, discussion of the assigned readings, exploration of importance and confidence in applying those skills in practice, and elicitation of residents’ perceived barriers and strengths for implementation. Role modeling through role play by the faculty is used to introduce the skills for each week.

Vignettes used for role-play are representative of common clinical presentations (often from residents’ own practices) and are time limited (10–15 minutes) to simulate the actual counseling portion of clinical encounters. Each role-play is followed by a five-minute feedback session.

Residents interview two standardized patients (SP) each week with direct feedback from the SP and observing faculty. The SP cases have been designed to provide an opportunity to apply the specific counseling skills being covered each week of the rotation. In addition to the SP experience, residents are directly observed in their continuity clinics and receive feedback while seeing their own patients.

### Assessment

**Figure 1:**
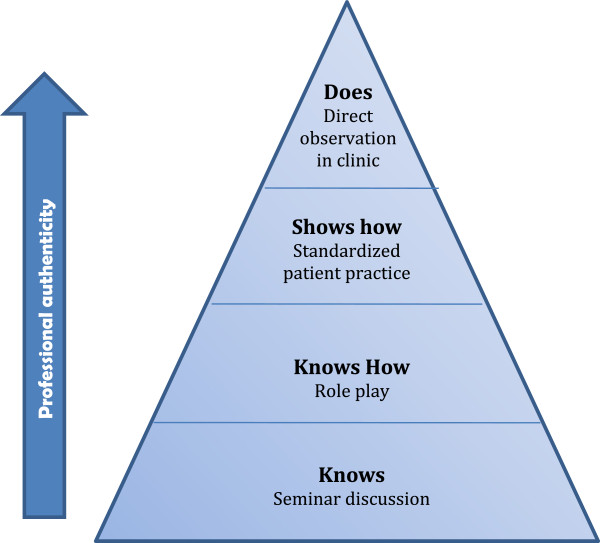
A Simple Model of Competence.

To determine the residents’ ability to translate concepts from the cognitive domain into behavior change in their clinical practice, we used an “authentic performance” assessment based on Miller’s pyramid model of competence [27] (See Figure 1). Residents’ acquisition and demonstration of skills are determined through direct observation of role-play, simulated practice with standardized patients and through observation of their continuity clinic with their own patients. Using a coaching technique [28], residents receive immediate feedback and opportunity to master each skill. To assess readiness to change, the residents rate themselves at the beginning and end of each week using importance and confidence scales regarding the implementation of the skills for that week. Importance and confidence scales are interviewing techniques used in motivational interviewing to assess and promote self-efficacy.

Thirty-nine residents have completed the curriculum to date. All residents have completed the rotation and have, based on the assessment previously described, demonstrated competency in the skills taught during the rotation. On average, residents’ importance and confidence scores were collectively higher than before being exposed to the curriculum. Finally, residents have subjectively reported high satisfaction with the rotation. This educational project was presented to the Institutional Review Board (IRB) and exempted from review.

## Results and discussion

In the style and spirit of MI, we present the following as general reflections and ideas for consideration and invitation to dialogue. The curriculum presented in this paper is an innovative working model for thinking about change in practice through the lens of an evidence-based approach to teaching, learning, and application. While MI has been applied broadly to various clinical populations and settings, it has not, according to our review of the literature, been applied as a model for teaching and facilitating behavior change among learners.

Feedback from our learners over the past several years is indicative of a positive shift in self-efficacy and confidence applying critical primary care counseling skills and counseling for health behavior change. In addition to residents self-report, our direct clinical observations corroborate the application of these skills in practice. Anecdotally, residents have verbalized an interest in export what they are learning to their psychiatry resident colleagues as well as involving them in the rotation experience.

The observed trend toward improved resident confidence/self-efficacy is promising, yet has not been systematically evaluated empirically. Clearly it is important to continue the process of self-reflection, taking in the voices of our learners with more formal, systematic evaluative methods. Future research should include skill retention over the course of training and video observation of residents in clinic with formal coding metrics to measure presence of and fidelity to the skills being taught. Additionally, measures related to patient satisfaction, behavior change, and other outcomes/indicators of clinical effectiveness should be included.

### Limitations

The curriculum described herein presents a small, albeit important step toward addressing the training gap in residency training programs. It is however a model based on limited self-report and informal observational data collected from one site.

## Conclusions

As we consider the broader implications and connections to what we have presented, we can’t help but feel we are “down a player.” We teach, model, and practice in a collaborative, team-based environment and expect our learners to do the same as they move toward independent practice. Yet, we find ourselves lacking the kind of multi-disciplinary, collaborative relationship with Psychiatry that would add a comprehensiveness and richness to the approach. Others have called for a “more expansive paradigm of care that conjoins the primary care physician to act within a larger network.” [29] The need for a shift in paradigm is not only important for provision of care but to the broader training environment of our learners. Future dialogue around these important issues of training and meeting the needs of the patients we serve is needed. Our hope is that there will be continued consideration of these topics and that discourse will lead to new paradigms shifts in how we prepare our learners to meet the demands that will be put upon them clinically as they move out of training.

## Endnotes

^a^ The longitudinal curriculum and additional information about residents’ self-evaluation and review of the rotation are available upon request.

## Abbreviations

PCPs: Primary care physicians; MI: Motivational interviewing; SP: Standardized patients; WHO: World Health Organization; RRC: Residency Review Committee; AAFP: American Academy of Family Physicians; WONCA: World Organization of Family Doctors.

## Competing Interest

No competing interests.

## Author’s contributions

ACT, MO, and DT participated in the curriculum design, implementation and evaluation. ACT and MO drafted the manuscript. All authors have read and approved the final manuscript.

## Pre-publication history

The pre-publication history for this paper can be accessed here:

http://www.biomedcentral.com/1472-6920/12/64/prepub
